# Gallium (III) Complexes Based on Aminobisphenolate Ligands: Extremely High Active ROP-Initiators from Well-Known and Easily Accessible Compounds

**DOI:** 10.3390/ijms232415649

**Published:** 2022-12-09

**Authors:** Badma N. Mankaev, Leyla F. Hasanova, Andrei V. Churakov, Mikhail P. Egorov, Sergey S. Karlov

**Affiliations:** 1 Chemistry Department, Moscow State University, 119991 Moscow, Russia; 2 N.D. Zelinsky Institute of Organic Chemistry, Russian Academy of Science, 119991 Moscow, Russia; 3 Institute of General and Inorganic Chemistry, Russian Academy of Science, 119991 Moscow, Russia

**Keywords:** gallium, ring-opening polymerization, lactide, ε-caprolactone, biodegradable polymers

## Abstract

We report herein the synthesis and full characterizations of the first examples of gallium complexes based on “privileged” aminobisphenolate ligands which are easily available. These complexes turned out to be extremely active in the ring-opening polymerization of ε-caprolactone even at room temperature and highly active in the ROP of L-lactide. The combination of factors such as the easy availability of these compounds and the supposedly low toxicity, together with the extremely high activity in ROP, allows us to consider these compounds as suitable for use on an industrial scale for the synthesis of biodegradable polymers for biomedical applications.

## 1. Introduction

Biodegradable polymers such as polylactide (PLA), poly(butylene succinate) (PBS), poly(ε-caprolactone) (PCL), polyglycolide (PGA), and poly(propylene carbonate) (PPC) during last three decades have become an attractive alternative to classic polyolefins in several fields of technology [1]. On the one hand, their physical and mechanical properties are often close to those of «classical» polymers based on alpha-olefins, on the other hand, their ability to decompose relatively quickly both in the environment and in living organisms determines two main directions of their use: these are various types of packaging and medical applications, such as, for example, suture material, etc. Among biodegradable polymers, PCL is one such material, which has received global attraction due to its biocompatibility, nontoxicity, and cost efficiency. It should also emphasize the presence of FDA approval for the internal medicinal application of PCL. These applications are controlled drug delivery systems, tissue-engineering scaffolds, the formation of artificial organs, nerve regeneration, and wound healing. Because PCL is semicrystalline as well as hydrophobic, it takes almost 3–4 years to degrade completely in vivo, making it a popular choice for long-term implants, bone tissue engineering, and slow-release medication administration [2,3,4].

Although the monomer of PCL, ε-caprolactone (ε*-*CL), is currently prepared from fossil resources in the industry, great success to date has been achieved in the synthesis of ε-caprolactone from renewable resources via the chemical treatment of saccharides [5]. The last makes PCL a fully sustainable polymer. In industry, PCL is made by ring-opening polymerization (ROP) of ε-caprolactone in presence of an initiator based on metal. Many main groups of metal and transition metal compounds have been reported as initiators for the ROP of ε*-*CL [6,7,8]. However, industrially used tin(II) bis-2-ethylhexanoic acid (tin octoate) in presence of alcohol is still the preferred catalytic system for the bulk polymerization of ε-caprolactone due to its high stability during the polymerization process and adequate activity in industry-reasonable polymerization conditions. Additionally, this initiator allows good control of polymerization and affords high-molecular-weight PCL with controlled properties [9,10]. At the same time, the use of tin octoate for PCL production for future medical applications is still in question due to the toxicity of tin compounds remaining in the polymer [11]. Thus, the synthesis of new initiators based on non-toxic metals, which produce PCL in a controlled manner with high molecular weight, and narrow molecular weight distribution due to the absence of side reactions from transesterification, remains relevant.

Amino bisphenols belong to the so-called “privileged ligands” and are easily available and inexpensive, giving them great potential as ligands for catalysts for a wide range of chemical transformations [12]. The simple synthetic approaches to these compounds allow for the introduction in the molecule of ligand different substituents. The steric and electronic properties of substituents, the nature of donor atoms in the ligand framework, and the size of the chelate formed with the metal atom determine the geometry and coordination mode adopted by metal complexes. The latter is very important for catalysis applications [13]. Aminobisphenolate complexes of main group elements (for example, Al [14,15,16,17,18,19], alkali metals [20,21,22], Ca [21], Mg [23]), transition metals (for example, Ti [19]) and lanthanides [24,25] have been reported to catalyze ROP reactions, but by now there is absolutely no information not only about catalysis but also about the synthesis of such gallium complexes in general. At the same time, gallium is a non-toxic metal if one may consider the concentrations of metal compounds that remain in the polymer during its synthesis [26], in addition, there are examples of the use of some gallium complexes in medicine [27], and gallium complexes of various structures (for example, diamido-ether dianionic ligands, (aminomethyl)phenolate monoanionic ligands, 8-quinolinolato monoanionic ligands, salan dianionic ligands, salen dianionic ligands, bis(imino)phenoxide monoanionic ligands) are active in ROP as initiators, [28,29,30,31,32,33,34,35,36,37] thus aminobisphenolate gallium derivatives should be considered as potentially promising ROP initiators and interesting targets for their synthesis and investigation of their behavior in ROP. 

In order to understand more about the structural chemistry of gallium complexes and the catalytic property of 13 Group elements complexes, we began to prepare gallium complexes bearing aminebisphenolate ligands and studied their catalytic property for the polymerization of ε*-*CL. All of them can catalyze the polymerization of ε*-*CL as well as L-lactide (L-LA) in absence of internal nucleophile (alcohol) with extremely high (ε*-*CL) and high activity (L-LA) to prepare PCL or PLA with narrow molecular weight distribution.

## 2. Results and Discussion

**Scheme 1:**
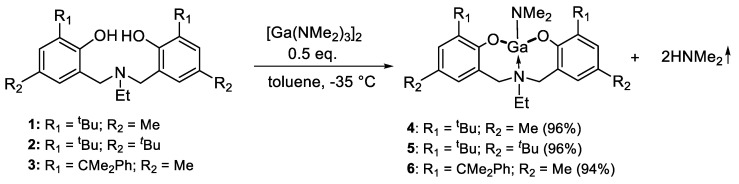
Synthesis of complexes **4**–**6**.

The room temperature reaction of amino bisphenols **1**–**3** (novel ligand **3** has been prepared according to a well-known literature procedure, see for example [19]) with equimolar amounts of dimeric tris(dimethyl)amide gallium (the molar ratio 1:0.5) led to monomeric gallium monoamides **4**–**6**, **EtN[CH_2_–(3-R^1^–(5-R^2^–)C_6_H_2_–2-O)_2_]_2_Ga–NMe_2_ (4, R^1^ = ^t^Bu, R^2^ = Me; 5, R^1^ = R^2^= ^t^Bu; 6, R^1^ = PhMe_2_C, R^2^ = Me)**, as white powders with high yields (Scheme 1).

All of those compounds were isolated as white solids and were characterized by spectroscopic studies as well as microanalyses. ^1^H and ^13^C{^1^H} NMR spectra of **4**–**6** (see Supplementary Materials Figures S2–S9 for details) are indicative of a highly symmetric species in solution, with two diastereotopic signals (two doublets of AB-system) corresponding to the methylene protons of N-CH_2_-aryl found around 3.0–3.5 ppm in C_6_D_6_. Of interest, there are two non-equivalent methyl groups in PhMe_2_C-substituents (two singlets 1.76 (6H) and 1.61 (6H) ppm in ^1^H NMR spectrum and two signals in ^13^C NMR spectrum) in **6** due to hindered rotation around Ar–CMe_2_Ph bonds.

**Scheme 2:**
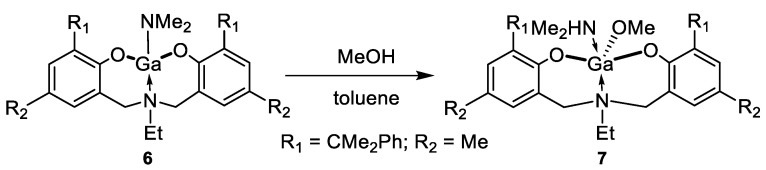
The plausible synthetic way for the formation of complex **7**.

**Figure 1:**
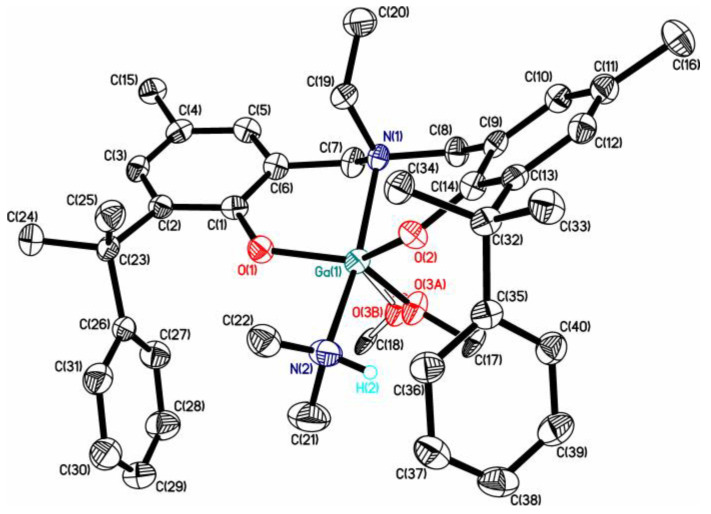
Molecular structure of **7**. Hydrogen atoms (except hydrogen atom on N(2)) and solvate molecule of toluene omitted for clarity. Selected bond Lengths (Å) and angles (deg) for **7**: Ga(1)-O(3A) 1.839(14), Ga(1)-O(1) 1.874(3), Ga(1)-O(2) 1.878(3), Ga(1)-O(3B) 1.89(2), Ga(1)-N(2) 2.091(5), Ga(1)-N(1) 2.119(4), O(3A)-Ga(1)-O(1) 118.7(4), O(3A)-Ga(1)-O(2) 108.6(4), O(1)-Ga(1)-O(2) 132.65(15), O(1)-Ga(1)-O(3B) 108.9(6), O(2)-Ga(1)-O(3B) 118.3(6), O(3A)-Ga(1)-N(2) 98.7(5), O(1)-Ga(1)-N(2) 88.54(18), O(2)-Ga(1)-N(2) 82.62(17), O(3B)-Ga(1)-N(2) 98.6(5), O(3A)-Ga(1)-N(1) 95.9(5), O(1)-Ga(1)-N(1) 89.12(15), O(2)-Ga(1)-N(1) 87.71(14), O(3B)-Ga(1)-N(1) 96.7(5), N(2)-Ga(1)-N(1) 164.46(17).

Unfortunately, we failed to grow crystals of **4**–**6** suitable for X-ray diffraction analysis. However, upon slow evaporation of the mother liquor remaining after recrystallization of **6**, a crystal of compound **7** was obtained, which has been studied by X-ray crystallography. Compound **7** is adduct of dimethylamine with EtN[CH_2_-(3-R^1^-(5-R^2^-)C_6_H_2_-2-O)_2_]_2_Ga-OMe. This compound was formed by the reaction of methanol, which apparently remained in trace amounts in ligand **3** after recrystallization of that from methanol during its purification and compound **6** (Scheme 2). The molecular structure of **7** is shown in Figure 1. The methoxy group was found to be disordered over two positions with 64/36 occupancies. Selected bond lengths and angles of **7** are listed in the Figure 1 caption.

Structure **7** exhibit a monomeric structure with a five-coordinate gallium center, including two six-membered amine phenolate metal-rings. The Ga–O_Ar_ bond lengths (1.874(3), 1.878(3) Å) as well as Ga–O_Alk_ (1.839(14) Å) are close to those found in related derivatives contained GaO_3_N_2_ fragment [31,33,37,38,39,40]. The Ga–N coordinative bond lengths (2.091(5) Å for NHC_2_, 2.119(4) Å for NC_3_) of the amine donor is close to those for related derivatives contained GaO_3_N_2_ fragment [40].

The important question is the substituents’ geometry around the Ga atom. The coordination polyhedron of the gallium atom in **7** can be described as either a distorted trigonal bipyramid with both N atoms in the apical positions and the three oxygen atoms in equatorial sites or a distorted tetragonal pyramid where N(1), N(2), O(1), O(2) atoms form pyramid base, O(3A)/O(3B) occupies the apical site. According to the approach of Addington et al. [41] and others [42], the assignment to one or another type of polyhedron can be made based on the value of the parameter τ which is applicable to five-co-ordinate structures as an index of the degree of trigonality, within the structural continuum between trigonal bipyramidal and rectangular pyramidal. For a perfectly tetragonal geometry τ = 0, while for a perfectly trigonal-bipyramidal geometry τ = 1. For **7** parameter τ has an intermediate value (0.53). Of interest, in similar Al aminobisphenolate complexes [18], where an Al atom bonded to two oxygen atoms (covalent bonds) and two nitrogen atoms (coordination bonds) parameter τ varies in the value range 0.53–0.77.

Compounds **4**–**6** were tested as catalysts towards the ROP of ε*-*CL and L-LA. The polymerizations of ε*-*CL were conducted in bulk at 80 °C. Compound **5** also was tested as the catalyst for the ROP of ε*-*CL at 25 °C and 100 °C; all the polymerizations of L-LA were conducted also under solvent-free conditions at 100 °C. In all studied cases, no external nucleophile such as alcohol has been used as a co-initiator. The polymerizations were monitored by taking aliquots at regular time intervals, which were analyzed using 1H NMR spectroscopy to determine the lactide conversion and by GPC (gel permeation chromatography) to determine the number of average molecular weight (Mn) and molecular weight distribution (PDIs, Mw/Mn). The polymerization results are summarized in Table 1.

Complex **5** tested was extremely active for the controlled polymerization of ε-caprolactone, as indicated by the monomer conversions and relatively narrow polydispersities of the PCL. It should be noted that the activity of initiator **5** (entry 6) is the highest (100% conversion, 15 min, 25 °C) among gallium complexes studied as initiators of the polymerization of caprolactone before. There are two comprehensive reviews of using gallium compounds for ester ROP published so far [7,8]. It is emphasized that heavier metal group 13 complexes based on gallium and indium have emerged as effective catalysts, but examples of catalysts containing these metals are still relatively rare and deserve further investigation. Nevertheless, indium compounds have been studied to a much greater extent than gallium compounds [7]. Perhaps, first of all, this is due to the fact that the Ga derivative of the salen type (O,N,N,O-type ligand with pentacoordinated Ga) is practically not active in ROP of lactide unlike the In complex of similar structure [37]. Additionally, the authors associated this poor activity with the electronic properties of the gallium atom. In addition, the gallium complex of N,O,N-type ligands with tetracoordinated Ga is practically not active in the ROP of CL [28]. However, the results of this work clearly demonstrate the particular importance of the structure of the ligand, which directly affects the activity of the initiator.

End-group analysis of the isolated PCL-200 (Table 1, entry 6) from the ^1^H NMR spectrum (see Supplementary Materials Figure S1 for details) indicates that the PCL chain is capped with one dimethyl amide and one hydroxyl chain end. This fact confirms that the “coordination-insertion” mechanism of ROP is implemented in this case [43]. Recently, the ability of complexes containing an amide moiety (M–NR_2_) to initiate ROP leading to polymers containing an amino-terminal group has also been demonstrated [44]. The M_n_ values of the obtained PCL samples (Table 1, entry 1–6) were lower than the expected values based on % conversion. This deviation likely implies that some transesterification reactions and/or a slow initiation step relative to propagation occurred. This trend is frequently observed in the ROP of the cyclic esters [45]. To demonstrate the capability of synthesizing PCL with a larger M_n_ and a well-controlled character, we accomplished ROP of ε*-*CL with [ε*-*CL]/[5] = 500 at 100 °C for 30 min (Table 1, entry 5). PCL with a large molecular weight, M_n_ (obsd) = 42,023 g mol^−1^ after the correction was obtained; its PDI in this case does not grow compared to the data obtained for a smaller ratio (Table 1, entry 2).

All synthesized complexes were also tested in ROP of L-LA (Table 1, entries 7–9) and showed slightly lower activity in this ROP compared to that for ε*-*CL. Previously, it was found that ε*-*CL usually has a higher polymerization rate in its respective homopolymerizations than L-LA [46]. The M_n_ values of the obtained PLA samples were very close to the expected values based on % conversion, which demonstrated the controlled character of L = LA polymerization. According to obtained NMR data, racemization did not occur during polymerization and pure poly-L-lactide is formed.

## 3. Materials and Methods

All reactions with air- and/or water-sensitive compounds were performed under a dry, oxygen-free argon atmosphere using standard Schlenk techniques. Solvents were dried by standard methods and distilled prior to use: toluene, *n*-hexane were refluxed under Na and distilled; ether, THF were stored under KOH, refluxed under Na/benzophenone and then distilled; methanol was refluxed under Mg and then distilled off. Starting materials were synthesized according to the literature procedures: 6,6′-((ethylazanediyl)bis(methylene))bis(2-(tert-butyl)-4-methylphenol) (**1**) [47], 6,6′-((ethylazanediyl)bis(methylene))bis(2,4-di-tert-butylphenol) (**2**) [48], [Ga(NMe_2_)_3_]_2_ [49]. C_6_D_6_ (dried over sodium), CDCl_3_ (dried with CaH_2_) and DMSO-d_6_ (dried over CaH_2_, distilled under reduced pressure, and stored under an argon atmosphere over molecular sieves) obtained from Deutero GmbH. ^1^H (400.13 MHz), ^13^C (100.61 MHz), were recorded on a Bruker Avance 400 or Agilent 400-MR spectrometers at room temperature (if otherwise stated). ^1^H and ^13^C chemical shifts are reported in ppm relative to Me_4_Si as external standard. Elemental analysis was performed using EuroEA-3000 (EuroVector, Pavia, Italy) instrument.

### 3.1. Synthesis of Ligand ***3***

In a flask, 4-methyl-2-(2-phenylpropan-2-yl)phenol (16.49 g, 73.1 mmol), 36% aqueous formaldehyde (5.97 mL, 70.20 mmol), 70% aqueous EtNH_2_ (2.50 mL, 39.0 mmol) and 15 mL of distilled water were placed. Solution was refluxed for 24 h, and then water was separated by decantation, and the mixture was dissolved in CH_2_Cl_2_ and washed with water. The organic layer was dried over MgSO_4_. Then, the volatile materials were removed under reduced pressure. The crude product was purified by recrystallization from methanol to afford the pure product. Compound **3** (12.3 g, 61%) was obtained as a white solid, m.p. 116–118 °C (from MeOH). ^1^H NMR (400 MHz, C_6_D_6_): δ (ppm) 7.28 (m, 4H; Ar*H*), 7.22 (d, *J* = 1.6 Hz, 2H; Ar*H*), 7.11 (m, 4H; Ar*H*), 7.03 (m, 2H; Ar*H*), 6.73 (d, *J* = 1.4 Hz, 2H; Ar*H*), 3.39 (s, 4H; NC*H*_2_Ar), 2.26 (s, 6H; C*H***_3_**Ar), 2.21 (q, *J* = 7.1 Hz, 2H; C*H***_2_**CH_3_), 1.68 (s, 12H; (C*H*_3_)_2_PhCAr), 0.67 (t, *J* = 7.1 Hz, 3H, CH_2_C*H*_3_); ^13^C NMR (100 MHz, CDCl_3_): δ (ppm) 153.15, 150.59, 136.21, 129.95, 129.07, 128.29, 127.36, 126.82, 126.50, 124.82 (Ar), 55.15 (N*C*H_2_Ar), 46.92 (*C*H_2_CH_3_), 42.45 ((CH_3_)_2_Ph*C*Ar), 30.22 ((*C*H_3_)_2_PhCAr), 21.49 (*C*H_3_Ar), 11.38 (CH_2_*C*H_3_); Anal. Calcd for C_36_H_43_NO_2_: C, 82.87; H, 8.31; N, 2.68; found: C, 82.95; H, 8.36; N, 2.76%.

### 3.2. General Synthesis of Ga Complexes (***4**–**6***)

A solution of the ligand (2.03 eqv.) in dry toluene was added dropwise to solution of [Ga(NMe_2_)_3_]_2_ (1 eqv.) in toluene with stirring at −35 °C. The resulting reaction mixture was stirred for 48 h. Then the volatile materials were removed under reduced pressure. The residue was recrystallized from toluene/*n*-hexane.

### 3.3. Synthesis of Complex ***4***

Prepared from 6,6′-((ethylazanediyl)bis(methylene))bis(2-(tert-butyl)-4-methylphenol) (**1**) (0.49 g, 1.24 mmol) and [Ga(NMe_2_)_3_]_2_ (0.27 g, 0.61 mmol). Yield: 0.59 g, 96%, white powder. ^1^H NMR (400 MHz, C_6_D_6_): δ (ppm) 7.27 (d, *J* = 1.9 Hz, 2H; ArH), 6.43 (d, *J* = 1.9 Hz, 2H; ArH), 3.33 (d, *J* = 12.5 Hz, 2H; NC*H*_2_Ar), 3.12 (d, *J* = 12.9 Hz, 2H; NC*H*_2_Ar), 2.78 (s, 6H; N(C*H***_3_**)_2_)), 2.42 (q. *J* = 7.0 Hz, 2H; C*H***_2_**CH_3_), 2.26 (s, 6H; C*H***_3_**Ar), 1.69 (s, 18H; C(C*H*_3_)_3_), 0.62 (t, *J* = 7.0 Hz, 3H, CH_2_C*H*_3_); ^13^C NMR (100 MHz, C_6_D_6_): δ (ppm) 159.07, 140.36, 129.52, 129.47, 126.63, 121.46 (Ar), 56.50 (N*C*H_2_Ar), 49.22 (*C*H_2_CH_3_), 42.61 (N(*C*H_3_)_2_), 35.69 (*C*(CH_3_)_3_), 30.40 (C(*C*H_3_)_3_), 21.37 (*C*H_3_Ar), 6.95 (CH_2_*C*H_3_); Anal. Calcd for C_28_H_43_GaN_2_O_2_: C, 66.02; H, 8.51; N, 5.50; found: C, 66.24; H, 8.60; N, 5.57%.

### 3.4. Synthesis of Complex ***5***

Prepared from 6,6′-((ethylazanediyl)bis(methylene))bis(2,4-di-tert-butylphenol) (**2**) (0.76 g, 1.57 mmol) and [Ga(NMe_2_)_3_]_2_ (0.32 g, 0.79 mmol). Yield: 0.85 g, 92%, white powder. ^1^H NMR (400 MHz, C_6_D_6_): δ (ppm) 7.59 (d, *J* = 2.4 Hz, 2H; Ar*H*), 6.69 (d, *J* = 2.4 Hz, 2H; Ar*H*), 3.40 (d, *J* = 12.9 Hz, 2H; NC*H*_2_Ar), 3.16 (d, *J* = 12.9 Hz, 2H; NC*H*_2_Ar), 2.78 (s, 6H; N(C*H***_3_**)_2_)), 2.42 (q. *J* = 7.0 Hz, 2H; C*H***_2_**CH_3_), 1.71 (s, 18H; C(C*H*_3_)_3_), 1.39 (s, 18H; C(C*H*_3_)_3_), 0.61 (t, *J* = 7.0 Hz, 3H, CH_2_C*H*_3_); ^13^C NMR (100 MHz, C_6_D_6_): δ (ppm) 158.87, 140.14, 139.79, 128.69, 125.54, 120.81 (Ar), 57.06 (N*C*H_2_Ar), 49.20 (*C*H_2_CH_3_), 42.58 (N(*C*H_3_)_2_), 36.04, 34.67 (*C*(CH_3_)_3_), 32.40, 30.44 (C(*C*H_3_)_3_), 6.92 (CH_2_*C*H_3_); Anal. Calcd for C_34_H_55_GaN_2_O_2_: C, 68.80; H, 9.34; N, 4.72; found: C, 67.62; H, 9.26; N, 4.64%.

### 3.5. Synthesis of Complex ***6***

Prepared from 6,6′-((ethylazanediyl)bis(methylene))bis(4-methyl-2-(2-phenylpropan-2-yl)phenol) (**3**) (0.81 g, 1.56 mmol) and [Ga(NMe_2_)_3_]_2_ (0.31 g, 0.78 mmol). Yield: 1.12 g, 94%, white powder. ^1^H NMR (400 MHz, C_6_D_6_): δ (ppm) 7.26 (m, 6H; ArH), 7.03 (m, 6H; ArH), 6.42 (s, 2H; ArH), 3.26 (d, *J* = 13.2 Hz, 2H; NC*H*_2_Ar), 3.03 (d, *J* = 13.2 Hz, 2H; NC*H*_2_Ar), 2.91 (q. *J* = 7.0 Hz, 2H; C*H***_2_**CH_3_), 2.50 (s, 6H; N(C*H***_3_**)_2_)), 2.28 (s, 6H; C*H***_3_**Ar), 1.76 (s, 6H; (C*H*_3_)_2_PhCAr), 1.61 (s, 6H; (C*H*_3_)_2_PhCAr), 0.61 (t, *J* = 7.0 Hz, 3H, CH_2_C*H*_3_); ^13^C NMR (100 MHz, C_6_D_6_): δ (ppm) 159.07, 152.86, 137.53, 129.67, 128.91, 128.47, 128.45, 126.63, 126.16, 121.29 (Ar), 57.19 (N*C*H_2_Ar), 48.39 (*C*H_2_CH_3_), 43.62 (N(*C*H_3_)_2_), 34.32 ((CH_3_)_2_Ph*C*Ar), 28.36 (*C*H_3_Ar), 21.42 ((*C*H_3_)_2_PhCAr), 6.49 (CH_2_*C*H_3_); Anal. Calcd for C_38_H_47_GaN_2_O_2_: C, 72.04; H, 7.48; N, 4.42; found: C, 72.15; H, 7.54; N, 4.46%.

### 3.6. Typical Polymerization Procedure in Bulk

All manipulations were performed under inert atmosphere. To the initiator **4** (0.0477 g, 0.098 mmol) *ε-*caprolactone (2.2257 g, 19.50 mmol) was added. The reaction mixture was heated at 80 °C for 15 min. The reaction was terminated by addition of MeOH (1.0 mL), evaporated, and purified by reprecipitation using CH_2_Cl_2_ as solvent and methanol as a non-solvent. The polymer obtained was dried in vacuum.

### 3.7. Single Crystal X-ray Diffraction Studies

Crystal data for **7**: C_46_H_59_GaN_2_O_3_, *F*_w_ = 757.67, monoclinic, *a* = 13.1068(6), *b* = 22.1139(11), *c* = 14.1810(8) Å, *β* = 95.663(2)°, *V* = 4090.2(4) Å^3^, space group *P*2_1_/n, Z = 4, *D*_c_ = 1.230 g/cm^3^, *F*(000) = 1616, *μ*(Mo*K*α) = 0.714 mm^−1^, colourless block with dimensions ca. 0.15 × 0.10 × 0.05. Total of 44,297 reflections (8026 unique, *R*_int_ = 0.169) was measured on a Bruker SMART APEX II diffractometer (graphite monochromatized Mo*K*α radiation, λ = 0.71073 Å) using *ω*-scan mode at 150 K. Absorption correction based on measurements of equivalent reflections were applied [50]. The structure was solved by direct methods and refined by full-matrix least-squares on *F*^2^ with anisotropic thermal parameters for all non-hydrogen atoms [51]. Amino hydrogen atom H2 was found from different Fourier syntheses and refined isotropically. All other H atoms were placed in calculated positions and refined using a riding model. Methoxy group was disordered over two positions with occupancy ratio of 0.714(7)/0.286(7). Solvent toluene molecule was found to be disordered over three sites. The final residuals were: *R*_1_ = 0.0715, *wR*_2_ = 0.1489 for 4509 reflections with *I >* 2*σ(I)* and 0.1489, 0.1774 for all data and 515 parameters. GoF = 1.004, maximum Δρ = 0.745 e × Å^−3^.

X-ray diffraction studies were performed at the Centre of Shared Equipment of IGIC RAS.

The crystallographic data for **7** have been deposited with the Cambridge Crystallographic Data Centre as supplementary publications under the CCDC number 2195341.

## 4. Conclusions

In conclusion, we have prepared the first examples of aminobisphenolate-based gallium complexes. These complexes are contained Ga-NMe_2_ fragments with the coordination number of the gallium atom equal to 4. We have demonstrated that these gallium complexes can be highly effective initiators for the production of PCL and PLA with relatively narrow dispersities and controllable molecular weights. This highlights the importance of the combination of metal/ligand in the rational design of initiators for the controlled ROP of cyclic esters.

## Figures and Tables

**Table 1:** Polymerization in bulk of *ε*-caprolactone and L-lactide.

Entry	Catalyst, [cat]	t, [min]	Conversion, [%]	M_n_ ^a^ _(calc)_, [g/mol]	M_n_ ^b^ _(exp)_, [g/mol]	PDI
*ε*-caprolactone (80 °C, [M]_0_/[cat] = 200:1)						
1	**4**	15	100	22,800	9172	1.80
2	**5**	15	100	22,800	15,407	1.51
3	**6**	15	100	22,800	28,538	1.47
*ε*-caprolactone (100 °C, [M]_0_/[cat] = 500:1)						
4	**5**	15	86	-	-	-
5		30	100	72,000	42,023	1.79
*ε*-caprolactone (25 °C, [M]_0_/[cat] = 200:1)						
6	**5**	15	100	22,800	12,270	1.38
L-lactide (100 °C, [M]_0_/[cat] = 200:1)						
7	**4**	15	75	-	-	-
8		30	100	28,800	28,620	1.36
9	**5**	15	79	-	-	-
10		30	100	28,800	21,595	1.17
11	**6**	15	71	-	-	-
12		30	100	28,800	21,834	1.26

^a^ Calculated according to the monomer conversion: M_n(calc)_ = M_w_(ε-CL) × ([ε-CL]_0_/[cat]) × (*conversion*) or M_n(calc)_ = M_w_(LA) × ([LA]_0_/[cat]) × (*conversion*). ^b^ The molecular weights and the PDI of the polymers were determined by GPC relative to polystyrene standards and multiplied by a correction factor of 0.58 for polylactide and 0.56 polycaprolactone.

## Supplementary Materials

The supporting information can be downloaded at: https://www.mdpi.com/article/10.3390/ijms232415649/s1.
