# Supplementary material for: Gallium (III) Complexes Based on Aminobisphenolate Ligands: Extremely High Active ROP-Initiators from Well-Known and Easily Accessible Compounds
Source: Int J Mol Sci. 2022 Dec 9;23(24):15649. doi: 10.3390/ijms232415649 (PMC9779430; doi:10.3390/ijms232415649)
Supplement: Supplementary file 1 [file ijms-23-15649-s001.zip › ijms-2053341-supplementary.pdf]

Supporting Information

Gallium (III) complexes based on aminobisphenolate ligands: extremely high active ROP-initiators from well-known and easily accessible compounds

Badma N. Mankaev,[1,2] Leyla. F. Hasanova,[1] Andrei V. Churakov,[3] Mikhail P. Egorov,[2] and Sergey S. Karlov[1,2,*]

[1]*Chemistry Department, Moscow State University, 119991 Moscow, Russia;*

[2]*N.D. Zelinsky Institute of Organic Chemistry, Russian Academy of Science, 119991 Moscow, Russia;*

[3]*Institute of General and Inorganic Chemistry, Russian Academy of Science, Moscow, 119991, Russia.*

**Correspondence: s.s.karlov@chemistry.msu.ru*

Table of Contents

Figure S1 ^{1}H NMR spectrum of PCL-200 (Table 1, entry 6) in $CDCl_3$. S2

Figure S2 ^{1}H NMR spectrum of **3** in C_6D_6. .... S3

Figure S3 ^{13}C NMR spectrum of **3** in C_6D_6. .... S3

Figure S4 ^{1}H NMR spectrum of complex **4** in C_6D_6. .... S4

Figure S5 ^{13}C NMR spectrum of complex **4** in C_6D_6. .... S4

Figure S6 ^{1}H NMR spectrum of complex **5** in C_6D_6. .... S5

Figure S7 ^{13}C NMR spectrum of complex **5** in C_6D_6. .... S5

Figure S8 ^{1}H NMR spectrum of complex **6** in C_6D_6. .... S6

Figure S9 ^{13}C NMR spectrum of complex **6** in C_6D_6. .... S6

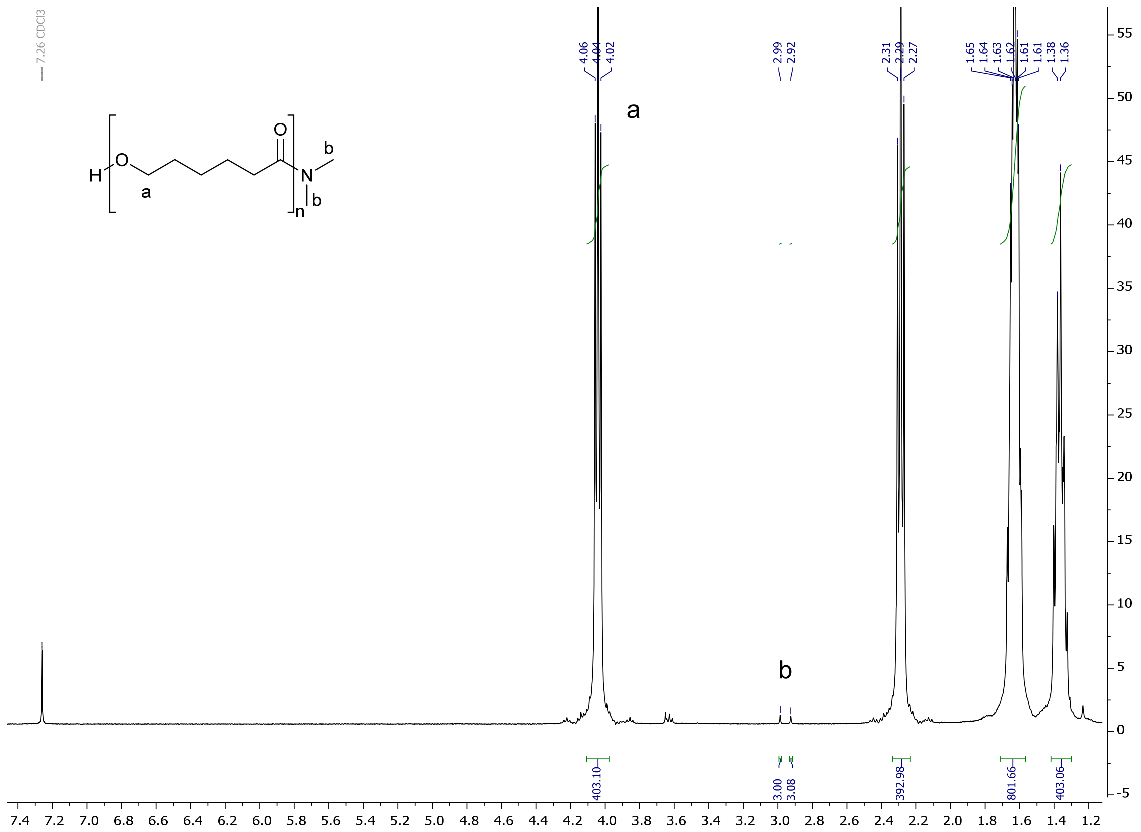

Figure S1 ^{1}H NMR spectrum of PCL-200 (Table 1, entry 6) in $CDCl_3$.

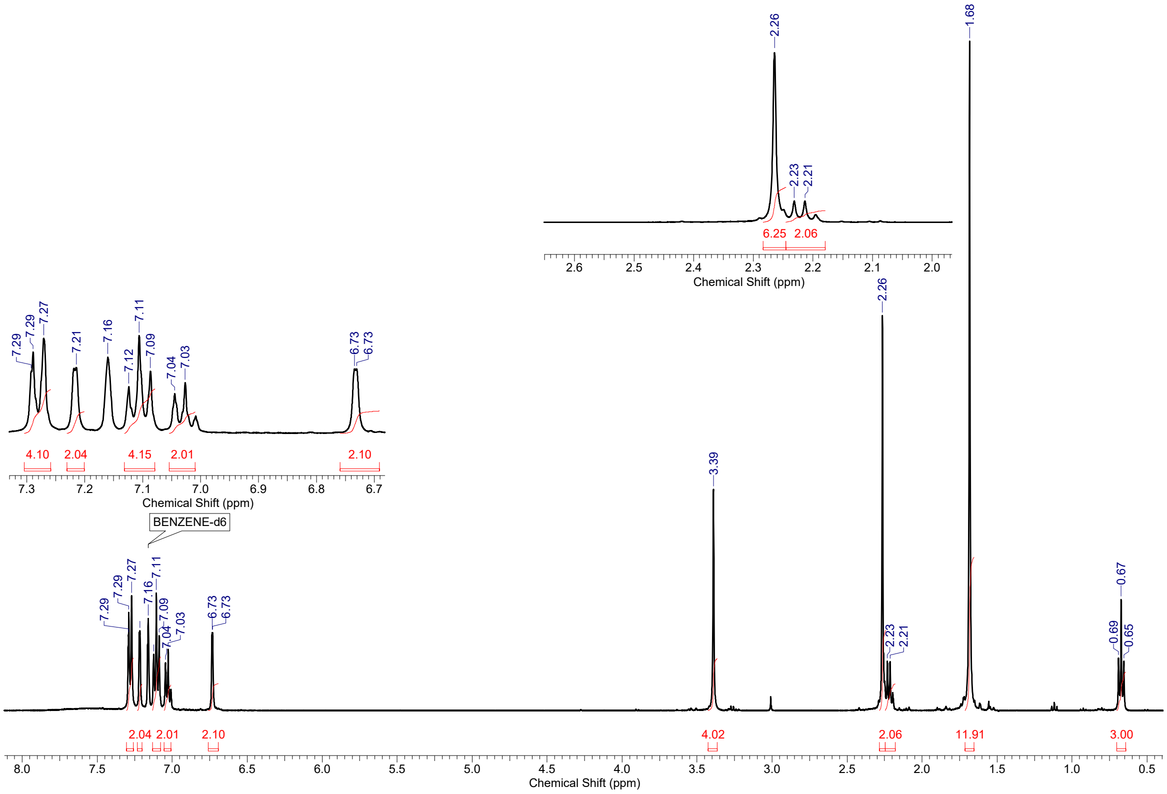

Figure S2 ^{1}H NMR spectrum of **3** in C_6D_6.

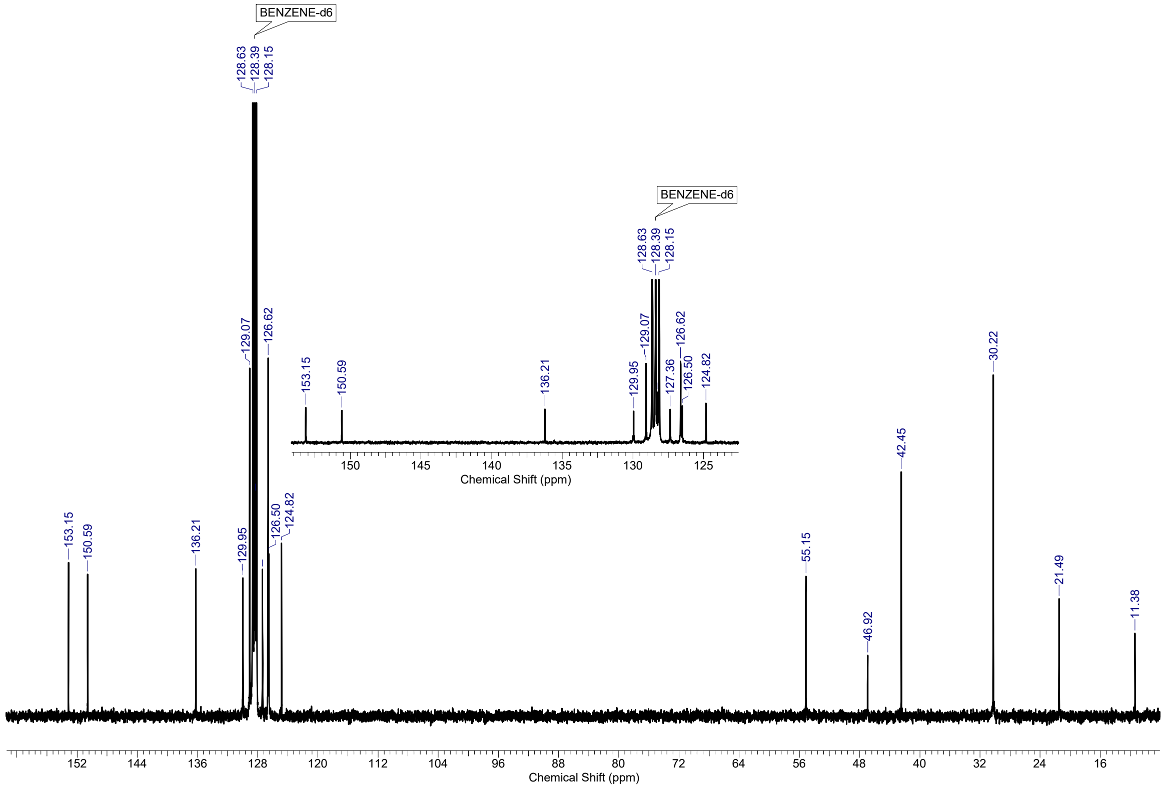

Figure S3 ^{13}C NMR spectrum of **3** in C_6D_6.

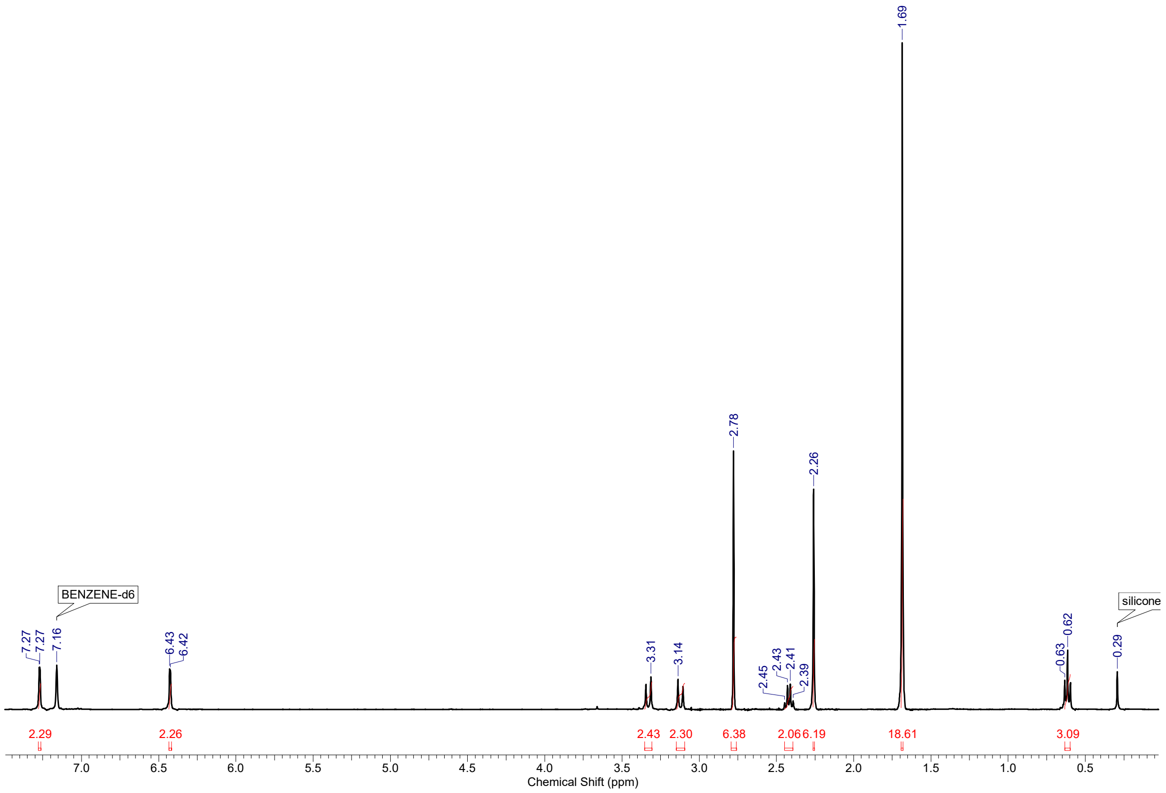

Figure S4 ^{1}H NMR spectrum of complex **4** in C_6D_6.

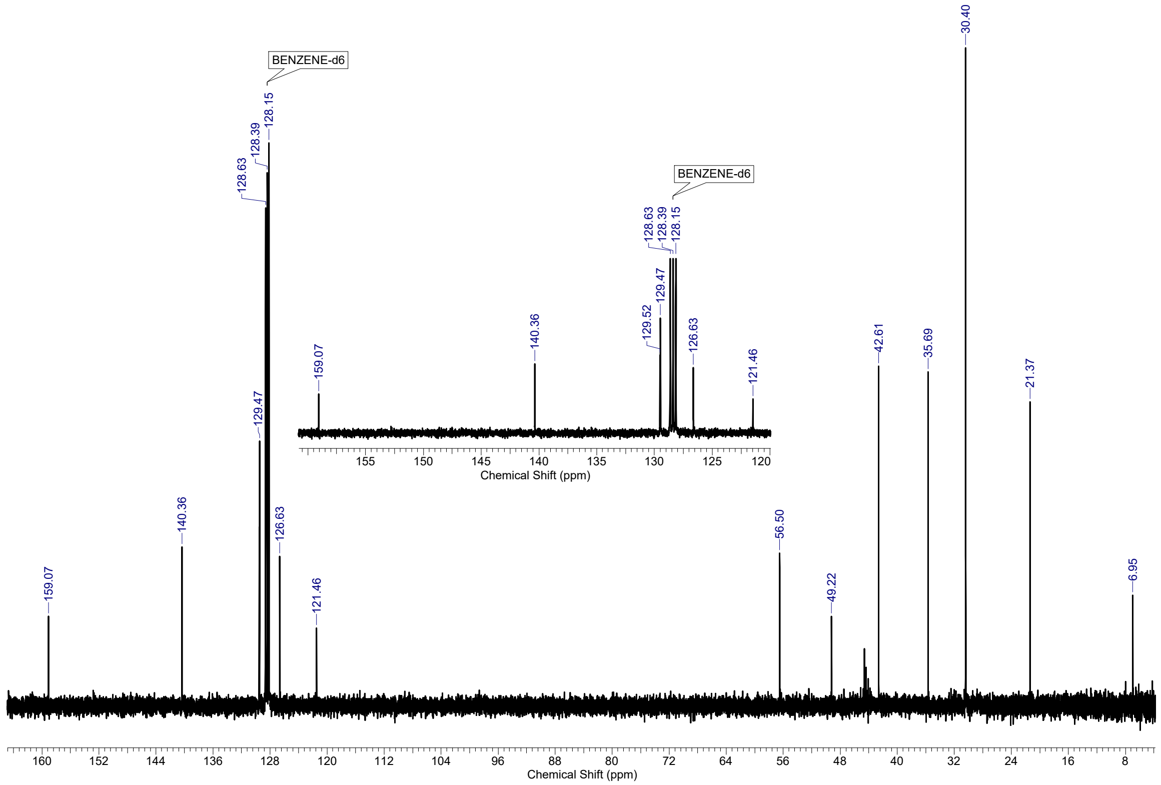

Figure S5 ^{13}C NMR spectrum of complex **4** in C_6D_6.

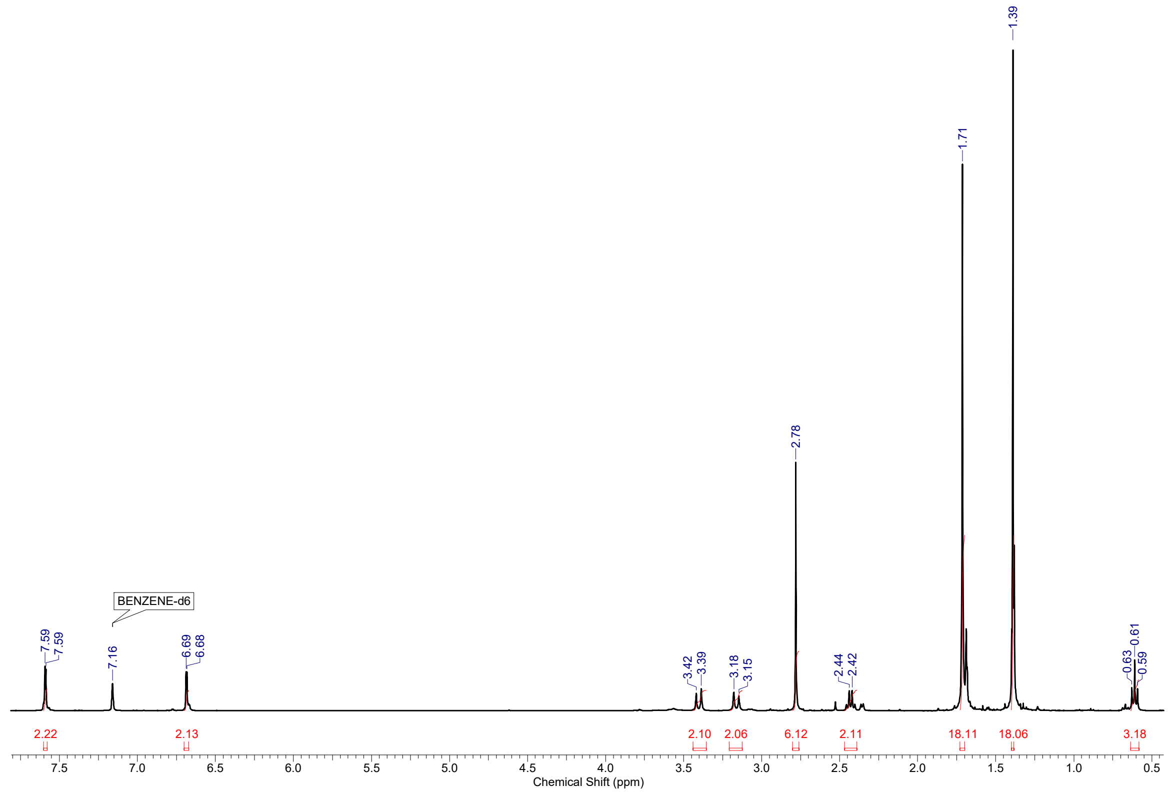

Figure S6 ^{1}H NMR spectrum of complex **5** in C_6D_6.

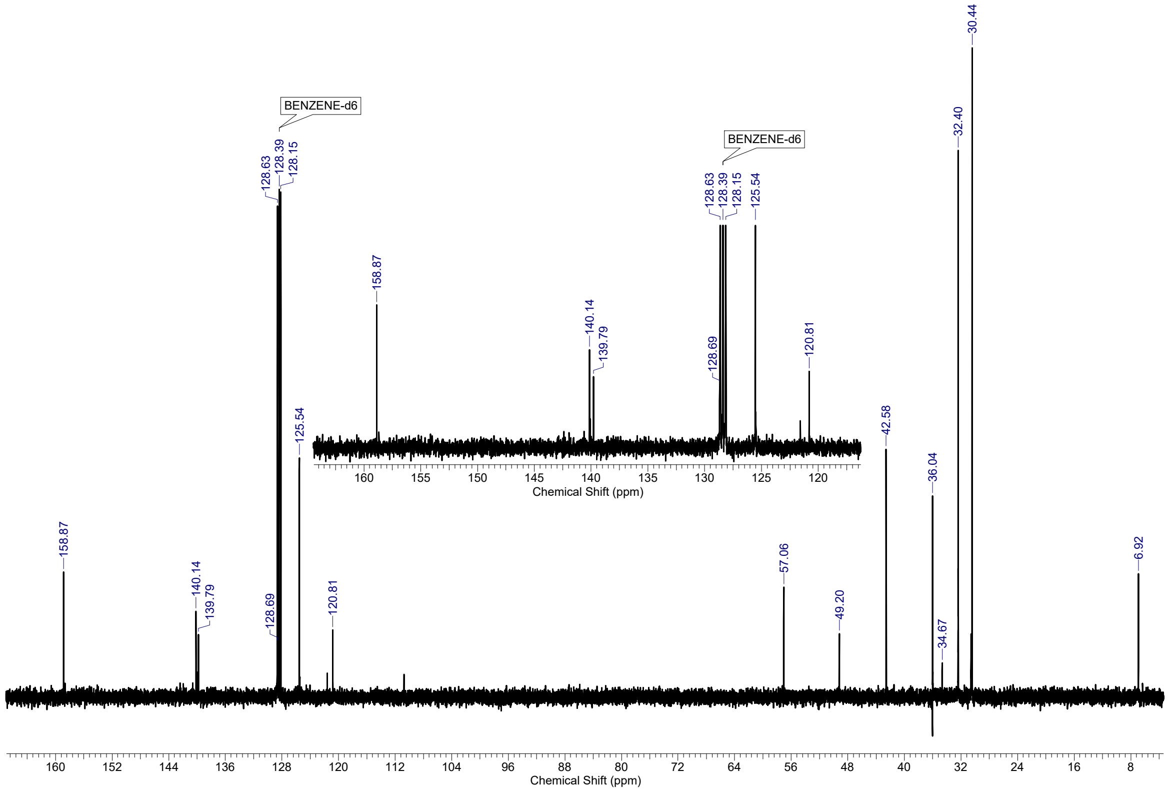

Figure S7 ^{13}C NMR spectrum of complex **5** in C_6D_6.

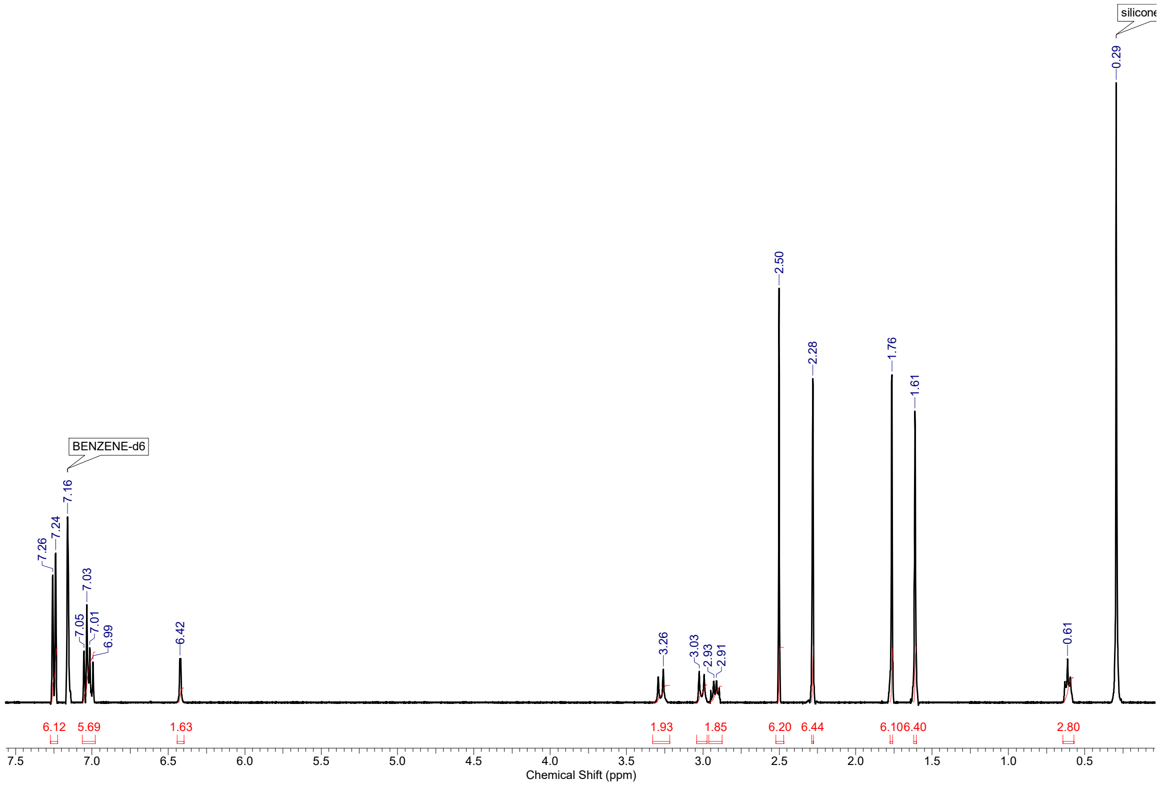

Figure S8 ^{1}H NMR spectrum of complex **6** in C_6D_6.

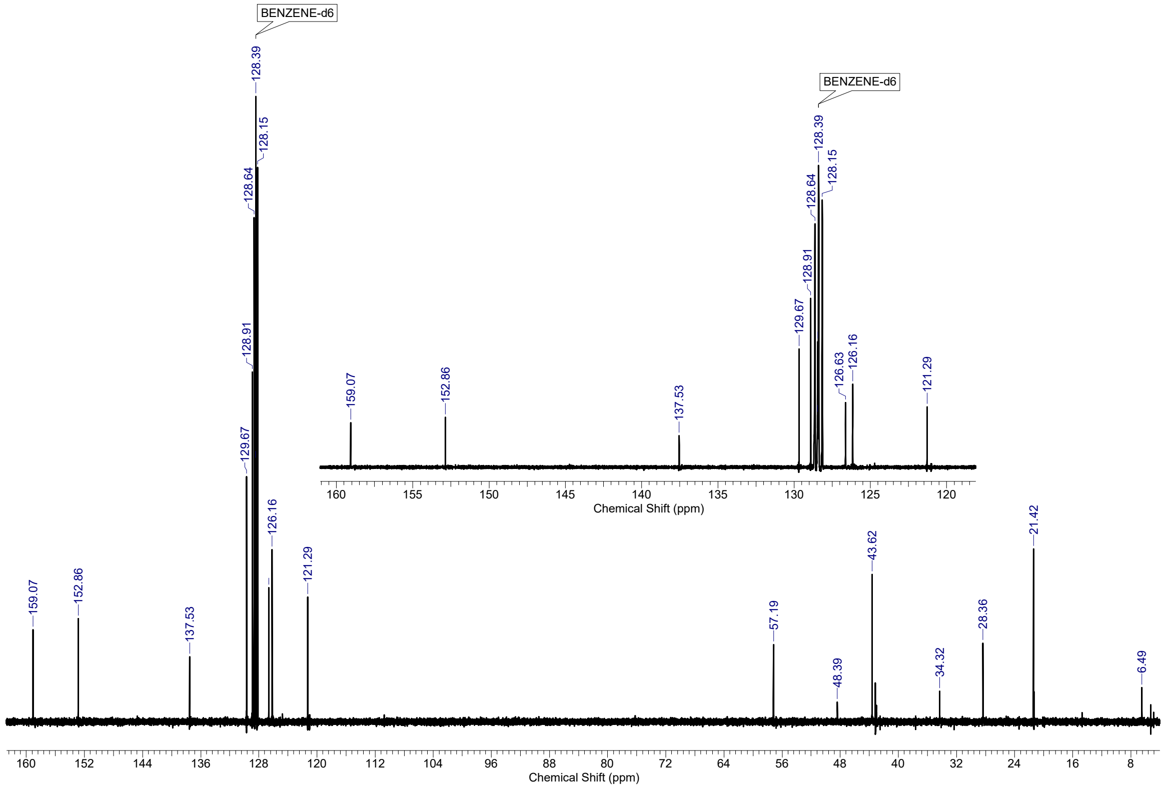

Figure S9 ^{13}C NMR spectrum of complex **6** in C_6D_6.
